# Reply to: The Physical Foundation of CSF‐IVIM

**DOI:** 10.1002/mrm.70621

**Published:** 2026-10-05

**Authors:** Yoshitaka Bito, Tomohiro Otani, Shigeki Yamada, Yoshiyuki Watanabe, Shigeo Wada

**Affiliations:** ^1^ Department of Diagnostic Imaging, Faculty of Medicine Hokkaido University Sapporo Hokkaido Japan; ^2^ Department of Mechanical Science and Bioengineering The University of Osaka Graduate School of Engineering Science Toyonaka Osaka Japan; ^3^ Department of Neurosurgery Nagoya City University Graduate School of Medical Science Nagoya Aichi Japan; ^4^ Interfaculty Initiative in Information Studies/Institute of Industrial Science The University of Tokyo Tokyo Japan; ^5^ Department of Radiology Shiga University of Medical Science Otsu Shiga Japan

1

We sincerely thank Dr. Levendovszky for the insightful commentary [1] on our recent publication about a theoretical interpretation of intravoxel incoherent motion for cerebrospinal fluid (CSF‐IVIM) [2]. We appreciate the comprehensive explanation regarding the value of our theoretical framework in neurofluids and the glymphatic system, as well as the thoughtful caveats regarding applicability limits when contrasting a simple theoretical framework with the high complexity of CSF physiology.

We strongly concur with the caution regarding the complexity of CSF physiology. Beyond the complexities of the brain parenchyma highlighted in the commentary [1], the boundaries of CSF spaces and their temporal dynamics have drawn substantial interest and warrant careful interpretation. For instance, the perivascular space (PVS) features complex CSF motion driven by cardiac, respiratory, and vasogenic motions, as investigated with dynamic diffusion‐weighted imaging [3] and selective CSF‐T2‐prepared readout with acceleration and mobility‐encoding (CSF‐STREAM) [4]. Furthermore, the perivascular subarachnoid space (SAS) surrounding large arteries in the SAS exhibits distinct CSF motion compared to other SAS regions, as investigated with CSF‐STREAM [4, 5]. In addition to these diffusion‐weighted techniques, other MRI techniques have been developed to investigate efflux as well as influx. Using spin‐labeling techniques, water exchange from tissue to CSF is assessed with magnetization transfer indirect spin labeling [6], while CSF drainage via meningeal and perivascular pathways is assessed with time‐spatial labeling inversion pulse [7]. Global and slower water transport and exchange are further assessed using stable isotope ^17^O‐labeled water [8, 9]. These techniques, alongside non‐MRI modalities such as high‐spatial‐resolution fluorescence imaging [10, 11], provide crucial insights into complex CSF physiology and warrant more sophisticated frameworks for CSF‐IVIM. Another essential direction of research on CSF dynamics analyzed using diffusion‐weighted techniques involves isolating advection from pseudorandom motion because advection is more directly linked to molecular transport. Indeed, under defined model assumptions, diffusion‐time dependency has been utilized to help distinguish advection (ordered motion) and diffusion (disordered motion) [12] based on a fundamental mathematical framework [13]. However, as emphasized previously, such a separation technique should be applied with caution to complex compartments such as the parenchyma, PVS, or meningeal drainage pathways.

We envision a bi‐directional research cycle: an inductive approach, where empirical findings from rapidly advancing techniques outlined above expand theoretical frameworks to accommodate complex CSF physiology; coupled with a deductive approach, where these enhanced frameworks guide interpretation, hypothesis formulation, and technical developments to further investigate CSF physiology. Ultimately, we share Dr. Levendovszky's enthusiasm for the rapid progress in the investigation of neurofluid dynamics and emphasize the value of a mathematical explanation to understand CSF dynamics.

## Funding

This work was supported by Japan Society for the Promotion of Science (26K03276, 25K15857, 25K03452).

## Conflicts of Interest

The authors declare no conflicts of interest.

## Data Availability

Data sharing not applicable to this article as no datasets were generated or analyzed during the current study.
